# Effects of menopause in a mouse model of Alzheimer’s disease and comorbid metabolic disease

**DOI:** 10.1002/alz.089245

**Published:** 2025-01-03

**Authors:** Charly Abi‐Ghanem, Abigail E Salinero, Richard D Kelly, Krystyna A Rybka, Olivia J Gannon, Kasey M Belanger‐Mayer, Christina A Thrasher, Emily Groom, Rachel Smith, Heddwen L Brooks, Damian G Zuloaga, Kristen L Zuloaga

**Affiliations:** ^1^ Albany Medical College, Albany, NY USA; ^2^ State University of New York at Albany, Albany, NY USA; ^3^ Tulane University School of Medicine, Tulane, LA USA

## Abstract

**Background:**

About two‐thirds of those with Alzheimer’s disease (AD) are women, most of whom are post‐menopausal. Menopause accelerates the risk for dementia by increasing the risk for metabolic, cardiovascular, and cerebrovascular diseases. Mid‐life metabolic disease (e.g. obesity, diabetes, or prediabetes) is a well‐known risk factor for dementia. A high fat diet can lead to poor metabolic health in both humans and rodents. The goal of this study was to determine the effects of menopause and high fat diet on metabolic, cognitive, and pathological outcomes in the App^NL‐F^ knock‐in mouse model of Alzheimer’s disease.

**Method:**

To model menopause, we used an accelerated ovarian failure model (4‐vinylcyclohexene diepoxide, VCD). This ovary‐intact model is more clinically relevant than an ovariectomy model, as mice go through a perimenopausal period. At 3 months of age, App^NL‐F^ mice were administered VCD or vehicle (oil) and then placed on either a control diet (10% fat) or a high fat diet (HF; 60% fat) and maintained on the diets until 10 months of age.

**Result:**

Menopause led to metabolic impairment (weight gain and glucose intolerance) and further exacerbated obesity in response to a high fat diet. Menopause had independent effects on some serum metabolic health biomarkers (insulin) and synergic effects with HF diet on other markers (glucagon). An interaction between HF diet and menopause led to an impaired episodic‐like memory. When examining the underlying pathology, we did not detect any changes in amyloid levels, however, we observe changes in neurogenesis and microglia response.

**Conclusion:**

Menopause and HF diet have independent and synergistic effects on metabolic, cognitive and pathological aspects of AD. This work highlights the need to model endocrine aging in animal models of dementia and will contribute to further understanding the interaction between menopause and metabolic health in the context of AD.

